# Experimental Investigations of Assessment of Acute Toxicity of Drilling Mud

**DOI:** 10.3390/toxics12100700

**Published:** 2024-09-27

**Authors:** Arstan Mamyrbayev, Saule Bermagambetova, Kuanysh Baytenov, Zhanat Komekbay, Laura Sakebayeva, Umit Satybaldiyeva, Gulmira Yerimbetova, Kulyash Zhilisbayeva

**Affiliations:** 1 Department of Hygienic Disciplines and Occupational Diseases, West Kazakhstan Marat Ospanov Medical University, 68 Maresyev Street, Aktobe 030019, Kazakhstan; arstan.mamyrbayev@mail.ru (A.M.); sakebaeva69@mail.ru (L.S.); 2 Department of Histology, West Kazakhstan Marat Ospanov Medical University, 68 Maresyev Street, Aktobe 030019, Kazakhstan; kuanish-1996kz@mail.ru (K.B.); zhanat.ru@inbox.ru (Z.K.); 3 Department of Internal Medicine No. 2, West Kazakhstan Marat Ospanov Medical University, 68 Maresyev Street, Aktobe 030019, Kazakhstan; satybaldieva.u@mail.ru; 4 Department for Scientific Work, West Kazakhstan Marat Ospanov Medical University, 68 Maresyev Street, Aktobe 030019, Kazakhstan; erimbetovagg@mail.ru; 5 Department of Languages, West Kazakhstan Marat Ospanov Medical University, 68 Maresyev Street, Aktobe 030019, Kazakhstan; kulyash.rakhmetullaevna@gmail.com

**Keywords:** drilling mud, toxicity and hazard, mean lethal dose, survival, behavioral reactions, environmental protection

## Abstract

At present, the main technological stages of oil production related to drilling operations require the use of a wide variety of drilling mud, which has a complex, multicomponent chemical composition. The drilling mud used and the resulting drilling waste must be safe for human health and the environment. The toxicity and hazard of drilling mud at this point in time remain poorly understood scientific problems and require detailing and studying in toxicological terms. The real degree of hazard and toxicity of drilling mud can only be determined by an experimental method, since its composition, which changes depending on the nature of the technological process and its degree of depletion, is not constant, which can change the toxicological properties. In an experiment conducted on adult male rats, under conditions of a single intragastric injection of drilling mud, new data were obtained regarding the parameters of its toxicity and hazard. The use of a wide variety of methods for determining lethal doses of drilling mud, including the probit analysis method, made it possible not only to substantiate the mean lethal dose of drilling mud but also other parameters of toxicity and survival of animals in the experimental groups. Features of eating behavior and body weight dynamics and the nature of the behavioral reactions revealed by the number and duration of stands and frequency and duration of grooming also indicate the presence of dose-dependent effects.

## 1. Introduction

In the process of oilfield development, a wide variety of drilling mud (DM) is used in the process of drilling wells and a large amount of drilling waste is consequently generated. Drilling mud, drill cuttings, and drilling wastewater contain a wide range of components harmful to the environment [1,2,3]. The issue of the toxicity and hazard of drilling mud still remains poorly understood and controversial. The available literature studies on the toxicity of drilling mud and drill cuttings were carried out mainly on test objects such as bacteria and aquatic organisms [4,5,6], and studies performed on mammals are extremely few [7,8]. The toxicological characteristics of the drilling mud and the degree of hazard to the environment and workers are mostly unknown and are largely classified by the manufacturers.

As a rule, drilling mud has a complex, multicomponent composition, which must be safe for human health and the environment, and have the necessary toxicological information to support statements on the degree of negative impact on the surrounding flora and fauna and the health of the working population. Available publications [9,10,11,12] do not cover the full scope of the research problem in this direction; they are often superficial, descriptive in nature, and characterized by incomplete development.

For drilling wells, solutions based on water and hydrocarbons are used. Technological engineers, depending on the stages of drilling at depth, conditionally divide all categories of drilling mud into groups. Drilling mud used in the first stage of drilling consists mainly of a clay–water mixture, sometimes with some low-hazard additives. As the well deepens, solutions with additional additives are used [13,14,15], designed to adjust the properties of the solutions in accordance with the density and properties of the rocks being passed through. These additives (chemicals) can change the hazard class of drilling mud towards increased toxicity. However, each batch of prepared drilling mud is repeatedly recirculated and diluted with formation waters; drilling mud components diffuse into formations at depth and mix with drill cuttings, which can ultimately lead to a decrease in the toxicity of the drilling mud, and waste drilling mud often has lower toxicity levels than the original drilling mud [10].

The above data indicate that the true value of the degree of hazard and toxicity of drilling mud can only be determined by an experimental method since its composition is multicomponent and varies depending on its degree of depletion.

The most appropriate method for conducting the experiment is intragastric administration of the drilling mud since the drillers’ workplace, including their work clothes and skin, particularly on their hands, is primarily contaminated with drilling mud, which can be ingested per os. Moreover, intragastric administration of the test substance allows for a more detailed and specific assessment of the general toxic and specific properties of the drilling mud. In contrast, with inhalation exposure, only chemical substances in a gaseous state can enter the worker’s body, which excludes the possibility of directly evaluating the toxicological effects of the drilling mud itself.

The aim of this work was to study in an experiment on mature male rats the toxicity of drilling fluid through the determination of a variety of lethal doses, including LD_50_, behavioral and nutritional reactions, and the nature of the general toxic effect.

## 2. Materials and Methods

In this study on the acute toxicity of drilling mud, mature outbred male rats with body weights of 180–220 g were used. Randomization of animals was carried out, where an important criterion was the body weight of the animal. Differences in body weight in the study groups did not exceed 10%. A total of 6 groups were formed with 6 animals in each group: control (group 1) and 5 experimental groups. In the acute experience experiment, the experimental groups were divided as follows: Group 2 of experimental animals received a single 300 mg/kg dose of drilling mud; Group 3—600 mg/kg; Group 4—1200 mg/kg; Group 5—2400 mg/kg; and Group 6—4800 mg/kg. The drilling mud was administered intragastrically using a probe. The individual volume of the administered dose of drilling mud for each animal was calculated based on body weight values. Drilling mud samples were taken from the Zhanazhol oil field located in Western Kazakhstan.

We used atomic absorption spectroscopy (Agilent AA240FS, Santa Clara, CA, USA) to study the content of heavy metals in drilling fluid and drill cuttings, which were taken from well SIV No. 64013. The determination of the content of heavy metals in drilling fluid and drill cuttings was carried out by the Aktobe Regional Center for Sanitary and Epidemiological Expertise. Sample analysis was carried out on the basis of State Standard 26929-94 [16]. This document describes in detail the preparation of samples for mineralization to determine the heavy metals under study, as well as the equipment, materials, and reagents to be used. The limit of quantification (LOQ) of chemical elements using this technique was (ng/L): Cd—0.08, Cu—0.41, Pb—0.16, Zn—1.02, Mn—0.66, Cr—0.99, Co—0.14, As—0.41, and for geological samples, the LOQ indicators were as follows (mcg/L): Cd—0.4, Pb—6.0, Zn—0.5, Mn—0.2, Cr—1.0, As—5.0.

The animals were kept in vivarium conditions that met sanitary standards [17], with free access to water, food, and natural light, and ambient temperature from 20 to 22 °C. Research methods are based on the use of Guideline R1.2.31.56-13 «Assessment of the toxicity and hazard of chemical substances and their mixtures for human health» M. Rospotrebnadzor 2014, 286p. The requirements for conducting research on experimental animals in this Guide are identical to international OECD document TG No. 420 «Acute Oral Toxicity—Fixed Dose Procedure». The GOST 33216-2014 guide to the care and maintenance of laboratory animals, rules for keeping and caring for laboratory rodents and rabbits, was also taken into account (adopted by the Interstate Council for Standardization, Metrology and Certification (protocol dated 22 December 2014 N 73-П)).

Animals were deprived of food 12 h before administration of the test compound. After the introduction of drilling mud, the animals were continuously monitored for their general condition, identifying signs of toxicity and the number and time of death of animals during the entire observation period, as well as food and water consumption. Observation was carried out for 6 h on the first day, after 24 h, and on subsequent days of the experiment. The observation period for the animals was 14 days. The general condition of the animals, the condition of the coat and skin, and the intensity and nature of spontaneous motor activity were assessed. In the experimental animals, indicators of motor activity (duration and number of vertical stands) and the emotional component of behavior—the number of grooming episodes—were recorded. The duration of the stands was recorded for 5 min on the 1st, 7th, and 14th days of the experiment, while the number of stands was recorded over the course of 1 h on the 1st, 7th, and 14th days of the experiment. Body weight was recorded in all groups before drug administration and weekly during the entire observation period and expressed in grams; body weight gain was also determined. At the end of the experiment, euthanasia and biomaterial collection were performed.

Based on the results of the death of animals, toxic doses were calculated—LD_16_, LD_50_, LD_84_, LD_100_—as well as indicators of the potential acute toxic hazard (S, R). Methods for determining toxic doses of certain chemical substances are divided into 2 groups, methods allowing the determination of only mean lethal doses (group 1), which include the methods of Behrens [18], Kerber [19], and Pershin [19], and methods that make it possible to determine not only mean lethal doses but also their errors, as well as confidence limits of activity ratios (group 2), which include the probit analysis method according to Finney [20] and probit analysis according to Prozorovsky [21]. The probit analysis method is more accurate, but the extreme indicators (LD_16_ and LD_84_) are often biased. The most accurate method for calculating these indicators is the least squares method using probit analysis. However, it is not without its shortcomings; excessive bulkiness and a large number of complex calculations complicate the work of researchers. The use of the same number of animals in the control and experimental groups and equal intervals between doses of drilling mud makes it possible to adequately use all of the above methods for assessing the toxicity of drilling mud.

Using software to automate elements of the calculation of this method makes it possible to use it in widespread practice. We used the StatPlus Version v 7 and StatPlus 5 Pro Version 6 programs to process the research results. It should be noted that the software represents a set of special documents that make it possible to upload research results to the MS database Excel and calculate the required toxicity parameters. The use of this program, which includes Probit analysis—Finney’s method—and Probit analysis—Prozorovsky’s method—made it possible to obtain not only the entire range of toxic effects of drilling mud, the lower and upper limit of LD_50_, the standard error, but also the weighting coefficient and probit indicators. The model for calculating Cox proportional intensities, embedded in the StatPlus program, made it possible to calculate the time of death and survival of experimental animals.

The results of the obtained studies were processed using variation statistics. Differences between groups were assessed using the parametric Student’s *t*-test, taking into account the Bonferroni correction, with the data presented in the form M ± SD, where M is the arithmetic mean, SD is the standard deviation, and also using the nonparametric Mann–Whitney U test, with the data presented in the form of median (Me) and lower and upper quartiles (Q 1, Q 3). Qualitative variables were analyzed using Pearson’s chi-square test. Correlation analysis was carried out using Spearman’s rank correlation coefficient. Quantitative parameters are presented as the mean value (M) and 95% confidence interval (±95% CI), or as the median (Me) and interquartile range (25%; 75%). The critical level of significance when testing statistical hypotheses was *p* ≤ 0.05.

The research was carried out within the framework of the scientific project IRN AR19676915 «Development of toxicometric criteria for the hazard and toxicity of drilling mud and cuttings», funded by the Science Committee of the Ministry of Science and Higher Education of the Republic of Kazakhstan. The approval of the local commission on bioethics of the West Kazakhstan Medical University named after M. Ospanov NJSC dated 15 March 2023 was gained, under Protocol No. 3 (3/14).

## 3. Results

Based on the results of an experiment involving a single acute exposure to drilling mud, data on eating behavior, weight gain, and behavioral reactions were obtained. Information was also obtained on the parameters of the toxic effect of drilling mud, assessed using various methods for studying mean lethal doses intended to study the toxicity and hazard of certain chemicals.

Table 1 shows the dose-dependent effects of animal death in an acute experience experiment with a single intragastric injection of drilling mud. It has been established that a single injection of drilling mud at a dose of 300–1200 mg/kg does not lead to the death of rats; at the same time, when drilling mud was administered at a dose of 2400 mg/kg, the deaths of two animals were detected, and at a dose of 4800 mg/kg, three rats.

It has been established that the mean lethal dose LD_50_ of drilling mud is 2739.4 mg/kg (95% confidence interval: lower limit of LD_50_—1470.1 mg/kg; upper limit of LD_50_—6949.0 mg/kg); Beta Standard error—0.0002. The toxicity parameters of the drilling mud (mg/kg) are as follows: LD_10_—1045.4; LD_16_—1417.7; LD_84_—4061.0; LD_90_—4433.4; and LD_100_—4721.8.

**Figure 1:**
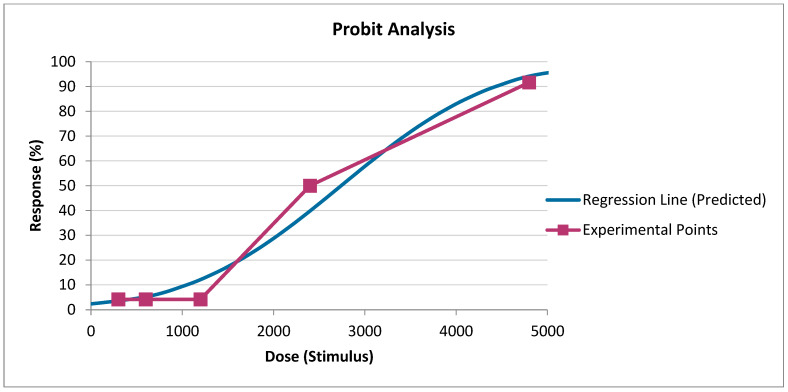
Determination of the mean lethal dose of drilling mud (probit analysis—Prozorovsky Method).

The LD_50_ values presented in Figure 1 indicate that the sigmoidal dose–response curves and their 95% confidence intervals quite convincingly highlight the dose dependence of drilling mud toxicity. At the same time, the plotted curves using logistic regression once again prove that drilling mud at doses of 2400 and 4800 mg/kg has a lethal effect on experimental animals.

Table 2 presents the parameters of drilling mud toxicity and death of experimental animals in an acute experience experiment with a single intragastric injection, calculated by probit analysis using the Finney method. It was once again shown that doses of drilling mud of 2400 mg/kg and 4800 mg/kg led to the death of two and three rats, respectively.

The mean lethal dose (LD_50_) of the drilling mud was 2400 mg/kg (95% confidence interval: lower—1937.8 mg/kg; upper—2972.4 mg/kg); Log10 [LD_50_]—3.3802, Standard Error—0.0474.

**Figure 2:**
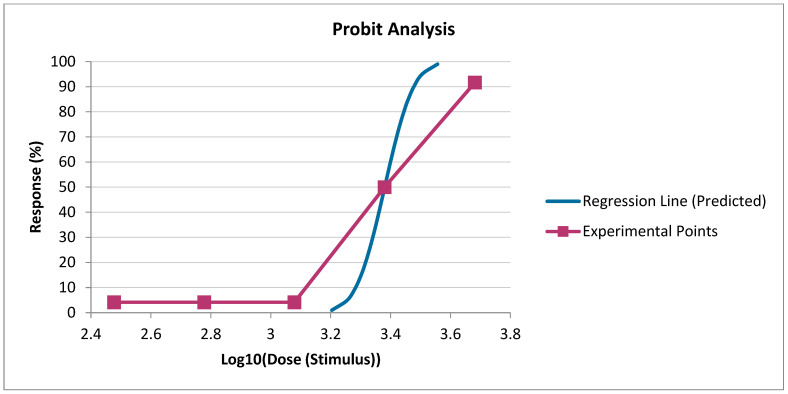
Determination of the mean lethal dose of drilling mud (probit analysis—Finney Method).

Figure 2 graphically presents the toxicity parameters of drilling mud, where the dose-effect curves reliably indicate that doses of drilling mud of 2400 mg/kg and 4800 mg/kg lead to the death of experimental animals.

Calculation of the mean lethal doses of drilling mud, with a single intragastric injection, according to the methods of Pershin, Kerber, and Behrens, showed the following: LD_50_ according to Pershin—1200 mg/kg; LD_50_ according to Kerber—3600 mg/kg; and LD_50_ according to Behrens—4055 mg/kg. The data obtained indicate a high dispersion of the mean lethal doses calculated by these research methods, which indicates their relative informativeness. Moreover, these methods do not allow the calculation of the entire range of toxicometric indicators, including the confidence interval and standard error value. The parameters determined by the probit method make it possible to obtain, to a greater extent, quite important and broad information about the toxicity and effect of drilling mud. Probit analysis is currently the most adequate and complete method for quantitative assessment of dose–effect relationships [22,23,24].

**Figure 3:**
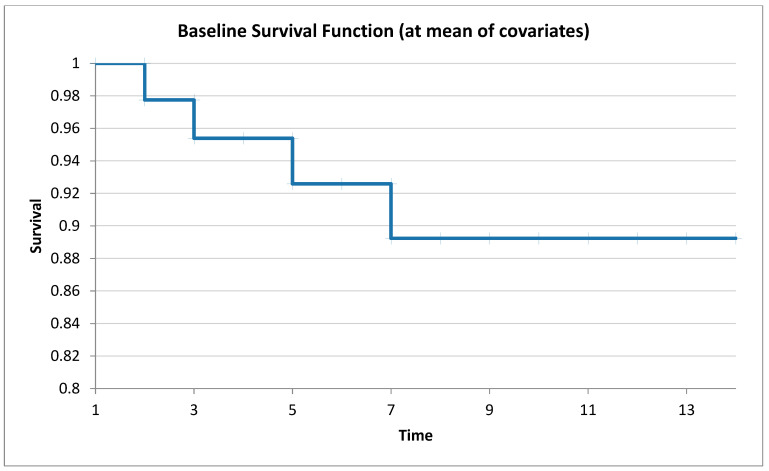
Acute survival rates (Cox method).

Using the Cox proportional intensity model, we established the survival of animals in an acute experience experiment with oral administration of drilling mud. Table 3 and Figure 3 show that the death of animals occurs on the 2nd, 3rd, 5th, and 7th days of the experiment. LD_50_ was 2800 mg/kg (lower level of the risk index—0.9997, upper level of the risk index—1.0010).

With a single intragastric injection of drilling mud into male rats in a dose range from 1200 mg/kg to 4800 mg/kg, signs of intoxication were recorded in the form of stiffness, tremor of the fore and hind limbs, impaired coordination of movement, and rapid breathing. In animals receiving drilling mud at doses of 2400 mg/kg and 4800 mg/kg, the inhibition of mobility and excitability gradually increased. By the end of the first day of observation, the animals moved little, reacted weakly to external stimuli, and assumed a lateral position. By the 14th day of the experiment, the fur of the experimental animals lost its shine and neatness and became dull. At the same time, attention was paid to changes in eating behavior and the dynamics of weight gain. Limiting indicators of the harmfulness of a chemical substance, which consisted of studying the behavioral reactions of animals, also indicated the peculiarities of the manifestation of the clinical picture of acute poisoning.

A single administration of DM at a dose of 2400 mg/kg led to the death of two rats: the first rat died on the 3rd day, and the second rat on the 7th day of the experiment. Meanwhile, the introduction of drilling mud at a dose of 4800 mg/kg was accompanied by the death of two rats on the 2nd day and one rat on the 5th day of the experiment. Intragastric single administration of DM in doses of 300 mg/kg, 600 mg/kg, and 1200 mg/kg did not lead to the death of experimental animals.

A study of feeding behavior, based on the consumption of food and water per day, was carried out throughout the entire experimental period from days 1 to 14. The amount of food consumed was measured by weighing the food on a scale and calculating the difference between the weight in grams of the food at the time of feeding and the remainder at the end of the day. Water consumption was assessed as the difference between the amount of water in milliliters in the sippy cup when it was filled and the remaining amount after 24 h. Measurements were carried out for the control and experimental groups of animals; The calculation of food and water consumed in grams and milliliters was correlated to 1 kg of animal body weight. It should be noted that in almost all experimental groups, there was a decrease in food and water consumption, and the most pronounced changes were observed in experimental groups where animals received large doses of drilling mud.

On the 1st day of the experiment, the amounts of food consumed per day in the experimental groups of animals receiving drilling mud at doses of 2400 mg/kg and 4800 mg/kg were 17.7 ± 0.7 g/kg (CI 15.4–20.1) and 15.7 ± 0.5 g/kg (CI 14.2–17.3), respectively. On the 7th day of the experiment in these experimental groups, feed consumption was, respectively, 18.2 ± 1.1 g/kg (CI 14.9–21.5) and 16.0 ± 13.1 g/kg (CI 11.7–20.3) per day, and on the 14th day of the experiment, it was 15.2 ± 0.5 g/kg (CI 13.7–16.8) and 14.7 ± 0.7 g/kg (CI 12.4–17.1), respectively. In the experimental groups, where animals were given a single intragastric injection of drilling mud in doses of 300 and 600 mg/kg, the dynamics of changes in feed intake were less pronounced. There were also certain changes in water consumption: in groups where animals received large doses of drilling mud (2400 mg/kg and 4800 mg/kg), water consumption per day decreased significantly on the 5th and 7th days of the experiment, with a subsequent increase in the amount of water consumed per day.

Table 4 shows the dynamics of changes in body weight of experimental animals under conditions of a single intragastric injection of drilling mud. It is noteworthy that on the 7th day of the experiment, a clear dynamics of a decrease in body weight of the experimental animals was revealed. The most significant changes were detected in those groups where animals received drilling mud in doses of 1200 mg/kg, 2400 mg/kg, or 4800 mg/kg. On the 14th day of the experiment, the revealed dynamics of changes in the weight of animals towards a decrease remained unchanged.

During the experiment, the duration and number of stands were studied in rats, the results of which are presented in Table 5. It should be noted that the duration of stands changed quite significantly downward in experimental rats that received drilling mud in doses from 1200 to 4800 mg/kg. Particularly significant differences were determined on the 7th day of the experiment, and by the end of the experiment (on the 14th day), this indicator tended to increase the duration of stands in almost all experimental groups of animals. In the experimental groups, the number of racks also tended to decrease compared to the control, and the most pronounced indicators were detected in groups of animals where rats received drilling mud in doses of 1200, 2400, or 4800 mg/kg. Again, as was the case with the study of stance duration, the number of stances was significantly reduced on day 7 of the experiment.

Grooming indicators (duration and frequency of grooming) also clearly demonstrated the dose dependence of this important parameter characterizing the behavioral reactions of experimental animals (Table 6). It was found that compared to the control, the duration of grooming is significantly reduced. Particularly low rates of grooming duration were found on the 1st and 7th days of observation of the rats. The lowest rates were found in animals receiving drilling mud at doses of 1200, 2400, or 4800 mg/kg. At the same time, on the 14th day of the experiment, no particularly significant changes were revealed, and, on the contrary, there was a tendency to restore the duration of grooming. In the experimental groups, especially on the 1st and 7th days of the experiment, where the animals received the highest doses of drilling mud, the frequency of grooming also tended to decrease. However, on the 14th day of the experiment, the frequency of grooming in the experimental groups, compared to the control, did not decrease and tended to increase.

Our research also examined the heavy metal content of drilling fluid and drill cuttings. The results showed that the lead content of the drilling fluid was 0.073 ± 0.006 mg/L and the arsenic content was 0.00014 ± 0.0004 mg/L. Meanwhile, the lead content in the drill cuttings was 13.22 ± 3.45 mg/kg and the manganese content was 77.6 ± 0.17 mg/kg. According to the engineering and technical personnel working at the oil production enterprise, the drilling fluid included bentonite clay (60 kg/m^3^), lime (8 kg/m^3^), ferrochrome lignosulfonate (12 kg/m^3^), carboxymethylcellulose (5 kg/m^3^), caustic soda (3 kg/m^3^), and BaSO_4_ (90 kg/m^3^).

## 4. Discussion

The toxicity and hazard of drilling mud remain poorly studied and require appropriate detailing and elaboration in toxicological terms. In the Republic of Kazakhstan, significant volumes of drilling are carried out—as a rule, by foreign companies—and a significant proportion of the chemical reagents and various materials used in drilling mud are imported from non-CIS countries. The toxicological characteristics of the drilling mud and the degree of hazard to the environment and workers are mostly unknown and are largely classified by the manufacturers. Of the regulatory documents for these products, there are only safety data sheets (MSDS), which occasionally provide only the maximum permissible concentration (MPC) values for the working area atmosphere.

The technological stages of oil production and refining require the use of a huge amount of chemical reagents and additives, so drilling mud has a complex, multicomponent composition. The specified reagents, drilling mud, and resulting drill cuttings must be safe for human health and the environment and have a final toxicological and hygienic composition that must mitigate the degree of negative impact of these multicomponent mixtures on the surrounding flora and fauna, and the health of workers.

At the same time, chemical reagents and additives used for oil production are quite diverse in their physicochemical properties [25,26,27]. In addition to demulsifiers, in polycomponent mixtures (drilling mud—DM, drilling cuttings—DC), a wide variety of corrosion inhibitors, scale inhibitors, hydrate formation inhibitors, depressants, polyacrylamides, polymers, aqueous solutions of chromium acetate, clay inhibitors, sodium and potassium polyacrylate, xanthine biopolymer, etc. are widely used [28,29,30].

The above data indicate that the true value of the degree of hazard and toxicity of drilling mud can only be determined by an experimental method, since its composition, which changes depending on the degree of depletion of the drilling mud, is not constant, which can change its toxicological properties.

As a result of our experiments on acute experience associated with a single intragastric administration of drilling mud to experimental animals, data were obtained on the toxicity and hazard of DM. Parameters of toxicity and hazard of DM have been established with the calculation of LD_50_ and other toxic doses—LD_10_, LD_16_, LD_84_, LD_90_, and LD_100_. The quantitative values we obtained for the acute toxicity of drilling mud after a single injection into the stomach allow us to classify DM as belonging to hazard class 4–5, according to the Hazard Classification of Chemical Substances and Their Mixtures according to Acute Toxicity Parameters [31].

Our results also show that the use of the probit analysis method (probit methods according to Finney and Prozorovsky) provides more reliable information on assessing the degree of toxicity of DM, which is supported by data from sigmoidal dose–effect curves. At the same time, other methods for estimating LD_50_ (Behrens, Pershin, Kerber methods) are not accurate and reliable. The results of our studies characterizing the potential and real hazard of acute fatal poisoning with DM are supported by data on the time of death and survival of experimental animals (Cox method). The time of death of the experimental animals occurred on days 2, 3, 5, or 7 of the experiment.

The study of eating behavior and the dynamics of body weight of experimental animals clearly showed that by the 7th and 14th days of the experiment, food and water consumption decreased, which directly affected the body weight of experimental animals, which also tended to decrease. The most pronounced changes were found in groups of animals that received the highest doses of drilling mud. The dynamics of the state of the central mechanisms for regulating eating behavior, the nature of the course of metabolism, and changes in digestive function play an important role in reducing the body weight of experimental animals under conditions of one or other intoxication [32,33]. The decrease in eating behavior and dynamics of weight gain in experimental animals under conditions of acute single exposure to DM is probably associated with the above mechanisms and malabsorption syndrome. It is known that the impact of chemicals on the digestive tract can manifest itself as inflammatory processes in the intestines and a decrease in the synthesis and excretion of bile acids in the liver necessary for the digestion and absorption of lipids, carbohydrates, and proteins [34,35].

The behavioral reactions we studied, along with the assessment of the general condition and appearance of the experimental animals, under conditions of acute single intragastric injection of drilling mud also prove the toxic properties of DM. The nature of the behavioral reactions, revealed by the number and duration of stands and frequency and duration of grooming, also indicates the presence of dose-dependent effects. In rats that received 2400 mg/kg or 4800 mg/kg doses of DM, the studied reactions were more pronounced than in animals that received 300 mg/kg or 600 mg/kg doses.

As the results of our research show, the toxicity of drilling fluid is largely determined by the content of heavy metals and other chemicals. The genesis of the toxicity and danger of heavy metals is quite wide and, firstly, lies in the production of reactive oxygen species, leading to increased lipid peroxidation of biomembranes, which ultimately leads to the development of cardiovascular diseases, neuronal damage, and kidney disease, and the risk of developing diabetes and cancer [36]. Lead, arsenic, and manganese are characterized not only by mechanisms of intensification of lipid peroxidation but also by adverse effects on the synthesis of protein and glutathione [37]. Our previous studies have convincingly proven the prevalence of imbalances in chemical elements among the adult population of Western Kazakhstan living in oil production regions [38]. At the same time, facts of contamination of the environment with heavy metals and their accumulation in biological media, which contribute to an increase in morbidity among the population, have been revealed [36]. The lime, ferrochrome lignosulfonate, carboxymethylcellulose, caustic soda, and barium sulfate contained in the drilling fluid under study have a pronounced general toxic and specific effect on living organisms [39,40,41,42].

Available literature data indicate that clayey-loaded drilling mud with a multicomponent composition, including calcium chloride, lime, and oil, is characterized by a fairly high hazard class (II Hazard Class), while waste drilling mud and drill cuttings based on these solutions belong to hazard classes III and IV, respectively [12]. Clay-loaded drilling mud, consisting of clay, barite, and chalk, and due to the low dissolvability of these weighting agents, has a hazard class of V. The waste generated from this DM is also non-hazardous. Clayey-loaded drilling mud also has very low toxicity rates, in which substances that are practically inert to dissolution (for example, iron-titanium dust (IKIMSO-TM)) are used as weighting agents. Clay emulsion drilling fluids, which contain oil, diesel fuel, and emulsion reagents, have fairly high toxicity levels, which are also transmitted to the liquid and solid waste generated from them. A number of authors express the point of view about the possibility of synergistic effects in polymer–clay drilling mud [43].

The toxicological assessment of components of drilling mud using biotesting methods [11] on such test objects as luminous bacteria Photobacterium fisher and algae Euglena gracilis showed that the toxicity of the drilling mud used was determined primarily by the degree of their saturation with sodium chloride, pH value, and the concentration of lignin-based reagents (the latter are acutely toxic substances). Lignin-containing reagents—alkaline slurry lignin and lingocel dissolved in a salt-saturated medium—had acute toxicity; however, as the concentration decreased, their toxicity dropped sharply. The presence of polymers such as polyacrylamide (PAA), carboxymethylcellulose (CMC), hypane, and some water-soluble salts, including those contained in formation waters, are of less importance from the point of view of toxicity. It is also important to know that the toxicity values of the used salt-saturated drilling mud under industrial operating conditions are not constant: the values are at a maximum for freshly prepared drilling mud and then decrease over time during its use [11].

It should be noted that the list of polymer reagents, widely used lubricants, corrosion inhibitors, clay inhibitors, a wide variety of surfactants, thickeners, detergents, and stabilizers includes more than 150 components of an organic or inorganic nature, all of which are widely used for the preparation of drilling fluids [9].

The results of the biotesting of drilling mud and waste based on drilling mud, using algae as a test culture, confirm the highly toxic nature of multicomponent drilling mud, especially that containing potassium bichromates and water-soluble salts in high concentrations. In the vast majority of the experiments conducted, the hazard classes of various solutions determined using daphnia and algae coincide or differ by no more than one gradation of hazard classes [12]. In clayey salt drilling mud, the nature of the toxicity depends mainly on the concentration of water-soluble salts.

At low salt concentrations, drilling mud, waste drilling mud, and drill cuttings belong to hazard class V (virtually non-hazardous). With an increase in salt concentration to 20% and above, and as the composition of the solution becomes more complex, the toxicity of the original drilling mud and waste drilling mud increases, while the toxicity of drill cuttings remains at the level of hazard class V. A similar pattern persists for clayey improved drilling mud. Solutions that include bentonite clay, small amounts of carbon-alkali reagent (CAR), and ferrochrome lignosulfonate (FCLS), as a rule, are not toxic, and toxicity does not appear in the liquid and solid waste generated from them. However, complicating such solutions by introducing into them, for example, potassium bichromates, even in small quantities, leads to a significant increase in toxicity. At the same time, the toxicity of their waste also increases [12].

Hydrocarbon-based drilling mud, and, accordingly, waste drilling mud and drill cuttings, are more toxic and hazardous than water-based drilling mud. As is known, the fractional composition of oil and petroleum products is characterized by a high content of aromatic hydrocarbons and polycyclic aromatic hydrocarbons. Heterocyclic analogs of polycyclic aromatic hydrocarbons are also common in oil, which causes a synergistic effect [44]. Numerous studies have convincingly proven the adverse toxic effect of oil and its components on ichthyofauna and ichthyoflora and experimental animals and humans [9,45]. Moreover, these substances are characterized by local, skin-irritating, skin-resorptive, and inhalation effects.

Using indicators of the degree of danger of a drilling fluid component (Ki), calculated as the ratio of the concentrations of waste components (Ci) with the coefficient of its degree of danger (Wi) for the environment, the hazard index of the drilling fluid and its grouping into hazard classes were calculated [43]. Group I included drilling fluids containing clays PBMA, PBMB, and PBMV, and CMC, CAR, oil, sulfanol, and hematite, which were classified as hazard classes IV and V. Group II included drilling fluids containing bentonite clays PBMA, PBMB, and PBMV, and lime, soda, technical lignosulfonates (LST), chromates, petroleum, sulfanol, and hematite. These drilling fluids are classified as hazard classes IV and V. Group III included drilling fluids containing bentonite clays PBMA, PBMB, and PBMV, and lime, soda, lignosulfonates (LST), chromates, petroleum, sulfanol, hematite, and condensed sulfite-alcohol stillage (СSAB), as well as FCLS. These drilling fluids contain a significant portion of components for which MPC values have not been established, namely, CMC, CAR, sulfanol, oil, СSAB, FCLS, soda, and chromates.

Often, the toxic effect of the studied multicomponent mixtures is due to the combined action of the chemical reagents included in their composition. Drilling mud and drill cuttings that contain petroleum products are more toxic. Considering the extremely diverse composition of drilling mud and drill cuttings, it is necessary to take into account the fact that the individual components included in their composition may be highly toxic and hazardous. Therefore, it is advisable to conduct studies in full, including their impact on ichthyofauna, mammals, and humans.

The experimental data presented in this article are part of a large scientific and technical project funded by a grant. In addition to these studies, we will also obtain data on the effects of drilling mud on peripheral blood parameters and detoxification enzyme activity, as well as the morphological and histological structure of internal organs and tissues. Furthermore, the results of studies focused on the detailed analysis of the chemical composition of the drilling mud will also be included. The comprehensive approach to evaluating the general toxic and specific effects of the drilling mud will provide a more substantiated scientific basis for extrapolating the experimental data to assess the safety of working conditions for people. The information presented will be very useful in predicting medical and environmental situations in oil production areas and organizing comprehensive measures to protect the health of the working population.

## 5. Conclusions

The research results presented in the present article point to the fact that drilling mud has a certain degree of toxicity and hazard, which largely depends on its chemical composition. The heavy metals we found in the drilling fluid (Pb, As, Mn) and the use of lime, ferrochrome lignosulfonate, carboxymethylcellulose, caustic soda, and barium sulfate in the technological process for producing drilling fluid indicate the specific etiological factors leading to the toxicity and hazard of the drilling fluid being studied.

It has been established that the mean lethal dose LD_50_ of drilling mud is 2739.4 mg/kg (95% confidence interval: lower limit of LD_50_—1470.1 mg/kg; upper limit of LD_50_—6949.0 mg/kg). The following parameters of drilling mud toxicity (mg/kg) were also determined: LD_10_—1045.4; LD_16_—1417.7; LD_84_—4061.0; LD_90_—4433.4; and LD_100_—4721.8.

Sigmoidal dose–response curves and their 95% confidence intervals quite convincingly emphasize the dose dependence of drilling mud toxicity. Curves constructed using logistic regression prove that drilling mud at a dose of 2400 and 4800 has a lethal effect on experimental animals. Calculations of mean lethal doses of drilling mud, with a single intragastric injection, according to the methods of Pershin, Kerber, and Behrens indicate a high dispersion of mean lethal doses, which indicates their relative informativeness but does not allow calculating the entire range of necessary toxicometric indicators. The parameters determined by the probit method make it possible to obtain, to a greater extent, quite important and broad information about the toxicity of the drilling mud and its confidence interval, and standard error value. The survival of animals in an acute experience experiment with oral administration of drilling mud showed that the deaths of animals occur on the 2nd, 3rd, 5th, and 7th days of the experiment.

The study of the general condition of the experimental animals, the dynamics of changes in body weight of the animals and the nature of food and water consumption, as well as the nature and type of severity of toxic manifestations, assessed by studying lethal doses and changes in behavioral reactions, indicate their dose dependence and indicate that the drilling mud has a certain degree of toxicity and hazard.

## Figures and Tables

**Table 1:** Parameters of toxicity of drilling mud and death of animals in an acute experience experiment (probit analysis—Prozorovsky Method).

Dose (mg/kg)	Number of Surviving Animals (N)	Probit (Y)	Weighting Factor (Z)
300	6	3.2680	1.5359
600	6	3.2680	1.5359
1200	6	3.2680	1.5359
2400	4	5.0000	5.0000
4800	3	6.3832	2.3503

**Table 2:** Parameters of drilling mud toxicity and animal death in an acute experience experiment (probit analysis—Finney method).

Dose (Stimulus)	Log10 [Dose]	Actual %	Probit %	N	Actual Count	Expected	Difference
300	2.4771	4.1667%	0.0000%	6	0.2500	0.0000	0.2500
600	2.7782	4.1667%	0.0000%	6	0.2500	0.0000	0.2500
1200	3.0792	4.1667%	3.5905 × 10^−5^	6	0.2500	0.0002	0.2498
2400	3.3802	50.0000%	50.0000%	4	2.0000	2.0000	4.1599 × 10^−9^
4800	3.6812	91.6667%	99.9964%	3	2.7500	2.9999	−0.2499

**Table 3:** Survival rate of animals in acute experience experiment (Cox method).

Baseline Survival Function (at Mean of Covariates)		
Time	Survival	Alpha
2.0000	0.9775	0.9775
3.0000	0.9539	0.9759
5.0000	0.9259	0.9706
7.0000	0.8924	0.9639

**Table 4:** Dynamics of body weight changes (mg/kg).

Observation Days	a—Control Group	Experience Group					
		b—300 mg/kg	c—600 mg/kg	d—1200 mg/kg	e—2400 mg/kg	f—4800 mg/kg	*p*—between Groups
Day 1	209.3 ± 7.9	210.7 ± 6.9	211.2 ± 7.7	209.5 ± 5.9	208.2 ± 5.7	205.7 ± 8	*p* = 0.800 *
Day 7	218.83 ± 7.2	207.7 ± 7	203.3 ± 7.9	199 ± 6.2	197.2 ± 4.7	195.8 ± 7.9	*p* < 0.001 *p*_ac_ = 0.006 *p*_ad_ < 0.001 *p*_ae_ < 0.001 *p*_af_ < 0.001
Day 14	224.3 ± 6.8	214.5 ± 6.7	210.8 ± 7.2	203.7 ± 5.9	201.5 ± 4.8	202 ± 7.7	*p* < 0.001 *p*_ac_ = 0.015 *p*_ad_ < 0.001 *p*_ae_ < 0.001 *p*_af_ < 0.001
*p*	*p* = 0.002	*p* = 0.002	*p* = 0.006	*p* = 0.002	*p* = 0.002	*p* = 0.002	

Note: *—differences in indicators are statistically significant according to Tukey’s test (*p* < 0.05).

**Table 5:** Indicators of motor activity.

Index	Control	Experience Group					R
		2	3	4	5	6	
DS5min—1 day (M ± SD)	154.7 ± 2.7	151.5 ± 22.7	153.5 ± 2.6	151.5 ± 3.4	144.5 ± 11.4	106 ± 8.7	<0.001 * p_1–6_ = 0.00 * p_2–6_ < 0.001 * p_3–6_ < 0.001 * p_4–6_ < 0.001 * p_5–6_ < 0.001 *
DS5min—7 day (M ± SD)	154.5 ± 1.9	149.2 ± 1.6	149.5 ± 1.2	112.7 ± 6.9	121.5 ± 5.1	134 ± 4.3	<0.001 * p_1–4_ = 0.001 * p_2–4_ < 0.001 * p_2–5_ < 0.001 * p_3–4_ < 0.001 * p_3–5_ < 0.001 * p_4–5_ < 0.022 * p_4–6_ < 0.001 * p_5–6_ < 0.001 * p_1–4_ = 0.001 * p_1–5_ = 0.001 *
DS5min—14 day (M ± SD)	154.2 ± 2.1	150.2 ± 2.8	151.5 ± 2.4	133.5 ± 9	144.5 ± 4.8	155.3 ± 2.1	<0.001 * p_1–4_ = 0.001 * p_1–5_ < 0.044 * p_2–4_ < 0.001 * p_3–4_ < 0.001 * p_4–5_ < 0.017 * p_4–6_ < 0.001 *
NS within 1 h—1 day (M ± SD)	22.8 ± 1.3	21.5 ± 1.5	21.5 ± 1.5	21 ± 1.1	19.8 ± 1.7	11.8 ± 3.3	<0.001 * p_1–6_ = 0.001 * p_2–6_ < 0.001 * p_3–6_ < 0.001 * p_4–6_ < 0.001 * p_5–6_ < 0.001 *
NS within 1 h—7 day (M ± SD)	22.2 ± 1.3	20.2 ± 1.3	19.7 ± 1.5	10.3 ± 1.5	12.2 ± 1.2	18.7 ± 0.6	<0.001 * p_1–3_ = 0.001 * p_1–4_ = 0.001 * p_1–5_ = 0.001 * p_1–6_ = 0.001 * p_2–4_ < 0.001 * p_2–5_ < 0.001 * p_3–4_ < 0.001 * p_3–5_ < 0.001 * p_4–6_ < 0.001 * p_5–6_ < 0.001 *
NS within 1 h—14 days (M ± SD)	22.2 ± 1.3	20.5 ± 1.2	21.7 ± 1.1	16 ± 0.6	14.5 ± 4.4	22.7 ± 0.6	<0.001 * p_1–4_ = 0.001 * p_1–5_ < 0.044 * p_2–4_ < 0.003 * p_2–5_ < 0.001 * p_3–4_ < 0.017 * p_3–5_ < 0.001 * p_4–6_ < 0.001 * p_5–6_ < 0.001 *

1—control group, 2—300 mg/kg, 3—600 mg/kg, 4—1200 mg/kg, 5—2400 mg/kg, 6—4800 mg/kg. Note: *—differences in indicators are statistically significant (*p* < 0.05).

**Table 6:** Grooming indicators.

Index	Control	Experience Group					R
		2	3	4	5	6	
GD sec—1 day (M ± SD)	15 ± 1.8	14.5 ± 1.9	15 ± 1.4	16.5 ± 1.6	13 ± 1.3	11.2 ± 1.2	<0.001 * p_1–6_ = 0.001 * p_2–6_ < 0.001 * p_3–6_ < 0.001 * p_4–5_ < 0.001 * p_4–6_ < 0.001 *
GD sec—day 7 (M ± SD)	14.7 ± 1.6	15.2 ± 1.2	13 ± 0.6	10.3 ± 1.2	12.7 ± 0.9	14 ± 1.1	<0.001 * p_1–3_ = 0.001 * p_2–3_ = 0.038 * p_2–4_ = 0.001 * p_2–5_ = 0.039 * p_3–4_ = 0.007 * p_1–4_ < 0.001 * p_4–5_ = 0.039 * p_4–6_ = 0.002 *
GD sec—14 days (M ± SD)	15.2 ± 1.5	14.7 ± 1.7	14.5 ± 1.4	1 3.2 ± 1.3	14.2 ± 0.9	16.7 ± 0.6	<0.001 * p_1–4 =_ 0.001 * p_2–4_ = 0.002 * p_3–4_ = 0.004 * p_4–5_ = 0.022 * p_4–6_ < 0.001 *
GF sec for 1 h—1 day (M ± SD)	3.3 ± 0.5	3 ± 0.6	3.2 ± 0.4	3.3 ± 0.5	2.2 ± 0.4	2.2 ± 0.4	<0.001 * p_1–5_ = 0.00 3 * p_1–6_ = 0.00 3 * p_3–5_ = 0.015 * p_3–6_ = 0.015 * p_4–5_ = 0.003 * p_4–6_ = 0.003 *
GF sec for 1 h—7th day (M ± SD)	3.3 ± 0.5	3 ± 0.6	3.2 ± 0.4	3.3 ± 0.5	2.2 ± 0.4	2.2 ± 0.4	<0.001 * p_1–6_ = 0.00 2 * p_2–5_ = 0.028 * p_2–6_ < 0.001 * p_3–6_ < 0.001 * p_4–5_ = 0.009 * p_4–6_ ≤ 0.001 *
GF sec for 1 h—14 days (M ± SD)	2.5 ± 0.5	3.33 ± 0.5	2.83 ± 0.7	2.5 ± 0.5	3.25 ± 0.5	3.5 ± 1.1	<0.001 * p_1–5_ = 0.003 * p_1–6_ < 0.001 * p_2–6_ = 0.011 * p_3–5_ = 0.020 * p_3–6_ = 0.001 * p_4–5_ = 0.003 * p_4–6_ < 0.001 *

1—control group, 2—300 mg/kg, 3—600 mg/kg, 4—1200 mg/kg, 5—2400 mg/kg, 6—4800 mg/kg. Note: *—differences in indicators are statistically significant (*p* < 0.05).

## Data Availability

Data are available from the corresponding author upon request.
